# Protein-Protein Interaction Network could reveal the relationship between the breast and colon cancer 

**Published:** 2015

**Authors:** Mona Zamanian-Azodi, Mostafa Rezaei-Tavirani, Sara Rahmati-Rad, Hadi Hasanzadeh, Majid Rezaei Tavirani, Samaneh Sadat Seyyedi

**Affiliations:** 1 *Proteomics Research Center, Shahid Beheshti University of Medical Sciences, Tehran, Iran*; 2 *Department of Cell and Molecular Biology, Faculty of Science, University of Tehran, Tehran, Iran.*; 3 *Cancer Research Center, Department of MedicalPhysics, Semnan University of Medical Science, Semnan, Iran*; 4 *Faculty of Medicine, Ilam University of Medical Sciences, Ilam, Iran*; 5 *Department of Medical Genetics, Tehran University of Medical Sciences, International Campus (TUMS- IC), Tehran, Iran*

## Abstract

**Aim:**

This study is aimed to elicit the possible correlation between breast and colon cancer from molecular prospective by analyzing and comparing pathway-based biomarkers.

**Background:**

Breast and colon cancer are known to be frequent causes of morbidity and mortality in men and women around the world. There is some evidence that while the incident of breast cancer in young women is high, it is reported lower in the aged women. In fact, aged women are more prone to colorectal cancer than older men. . In addition, many studies showed that several biomarkers are common among these malignancies.

**Patients and methods:**

The genes were retrieved and compared from KEGG database and WikiPathway, and subsequently, protein-protein interaction (PPI) network was constructed and analyzed using Cytoscape v:3.2.1 software and related algorithms.

**Results:**

More than forty common genes were identified among these malignancies; however, by pathways comparison, twenty genes are related to both breast and colon cancer. Centrality and cluster screening identified hub genes, including SMAD2, SMAD3, (SMAD4, MYC), JUN, BAD, TP53. These seven genes are enriched in regulation of transforming growth factor beta receptor signaling pathway, positive regulation of Rac protein signal transduction, positive regulation of mitochondrial outer membrane permeabilization involved in apoptotic signaling pathway, and positive regulation of mitotic metaphase/anaphase transition respectively.

**Conclusion:**

As there are numerous genes frequent between colorectal cancer and breast cancer, there may be a common molecular origin for these malignancies occurrences. It seems that breast cancer in females interferes with the rate of colorectal cancer incidence.

## Introduction

 Colon cancer accounts for the second most widespread fatal malignancy and with 30% inheritance bases in the world (1, 2). It manifests in the lower section of digestive system known as large intestine (colon) (3). Regular treatment for colon cancer comprise of surgery, chemotherapy and radiotherapy (1). An accumulation of mutations in tumor suppressor genes and oncogenes is the cause of cancer progression, which is a multistep process. This development requires many genetic alterations. The inactivation of the APC (adenomatous polyposis coli) pathway manifests the onset of the cancer. In addition, other genetic mutations, including APC, SMAD2, 4, TP53 and oncogenes (KRAS) promotes metastasis events. Furthermore, epigenetic factors can increase promoter alteration and result in expression deregulation of oncogenes and/or tumor suppressor genes (4). Breast cancer, on the other hand, is one of the most prevalent malignancies among women around the world (5). In the United States, about 14% of women’s cancers are diagnosed with breast cancer, which makes it as the second cause of cancer death (6). The early detection and appropriate treatment of this heterogeneous malignancy is required for decreasing the mortality rate. Suggested treatments for breast cancer comprise of chemotherapy, radiotherapy, immunotherapy or targeted therapies, and surgery (7). Some factors can increase the cancer risk. These factors include age, nulliparity, positive family history of breast cancer, and use of menopausal hormone therapy (5). Breast cancer is a complex genetic malignancy that diverse kinds of genetic and epigenetic alteration have been reported for this cancer trigger and development (8, 9). Somatic genetic mutation has a great involvement in this malignancy. The significant genes that play role in breast cancer progress are *BRCA1, RB1, TP53, PTEN, AKT1, CDH1, GATA3*, and *PIK3CA*. Mutation in these genes can lead to dysfunction of apoptosis, cell-cycle regulation, and transcription regulation. Other somatic genes that are implicated in signal transduction includes *APC, ARID1A, ARID2, ASXL1, BAP1, KRAS, MAP2K4, MLL2, MLL3, NF1, SETD2, SF3B1, SMAD4, *and *STK11*. Moreover, there other types of somatic genes that recently assigned with breast cancer pathogenicity are *ARID1B, CASP8, MAP3K13, MAP2K13, NCOR1, SMARCD1, *and *CDKN1B. * In addition, the presence or absence of some specific proteins including estrogen receptors (*ER*), progesterone receptors (*PR*), and human epidermal growth factor receptor-2 (*HER2*) (8). Breast and colon cancer manifest from the same type of tissue, known as epithelial which is a suitable tissue for tumor development (10). In addition, some effective common risk factors for breast and colon cancer include, obesity (11, 12), nutrition (calcium, vitamin D) (13), and physical activity (14). There is an indefinite link between fat and protein animal intake and increment risk of breast and colon cancer. Vitamin D, on the other hand, has some beneficial effect in decreasing the risk of breast and colon cancer. For colon cancer, it can cause lowering the probability of polyps and adenomas in the colon (15). There are several reports relative to either close relationship or differences between breast and colon cancer (16, 17). Recently, PPI networks attracted scientist’s attention due to its powerful ability in interpretation of biological phenomena of diseases (18). In this approach a set of candidate genes is located in a network including the relevant proteins. The two concepts; network properties and the role of each protein are informative findings for interpretation of investigated disease (19). PPI networks are prevalent in cancer research, however, studies have revealed interesting topological properties of PPI networks (20). Breast and colon cancer are investigated separately via PPI network analysis and more details of gene expression are reported (21, 22). In this research, close molecular relationship between breast and colon cancer is discussed and a new epidemiological glance is presented. 

## Patients and Methods

Common genes of breast and colon cancer for KEGG Pathway and WIKI Pathways were downloaded from websites (http://www.genome.jp /kegg/pathway.html and http://www.wikipathways.org/index.php/WikiPathways, respectively) and compared manually. Twenty genes were identified and candidate for this study. Uniprot accession number of selected genes was retrieved from (uniprot.org). The codes were used for further evaluations. For PPI network construction, the codes were searched against Proteomics Standard Initiative Common QUery InterfaCe (PSICQUIC), which is a plug-in in *Cytoscape v: 3.2.1.* Software that imports relevant information from public databases. MINT, Reactome-Fls, STRING databases were used for this topology visualization. By the application of Cytoscape as an open-source tool, interaction between molecules can be visualized. Additionally, other attributes can be integrated to these interactions (23). 

The Cytoscape platform actively supports the development of plug-in tools that extend the core functionality (23). Topological centralities (degree and betweenness centrality) were evaluated to distinguish the biological value of genes, pathways and clusters. The number of edges that are connected to a designated node is the degree. The high degree indicates the significance of the gene in biological interactions, known as hub. In addition, the number of shortest paths that pass through each node implies betweenness centrality value. The node size and color changes indicate the value of hub based on centrality parameters. As the circles get bigger and their color change from green to red, their value of the degree and betweenness centrality increase. This evaluation is accessible through Network Analyzer. This tool is a promising analyzer that performs inclusive evaluations of simple and complex topology features and presents it as graph algorithms. Many fundamental topological parameters can be computed by the use of this plug-in (24).  

Molecular complex detection (MCODE) is a useful method to identify clusters of highly connected nodes and computing relevant score. The score is computed based on the local density of each node in the graph (25). MCODE parameters include Node Score Cutoff: 0.2, K-Core: 2, and Threshold: 2 for each sub-network. Additional assessments were handed by using other algorithms. Clue Go is also a cytoscape software for gene ontology and pathway enrichment analysis. Different criteria are applied for annotation analysis of studied modules (protein complexes). These include Kappa statistic ≥ 0.4, enrichment (Right-sided hypergeometric test), and Bonferroni step down method for probability value correction (26). 

## Results

The Kyoto Encyclopedia of Genes and Genomes (KEGG), and WIKIPathway are the selected databases for pathway comparison between cancers of breast and colon. 

**Table 1:** A number of twenty common genes between breast and colon cancer derived from KEGG and WikiPathway

Gene Name	Protein Name	Uniport Accession ID	Chromosome Location
BAD	Bcl2-associated agonist of cell death	Q92934	11
SMAD2	Mothers against decapentaplegic homolog2	Q15796	18
SMAD3	Mothers against decapentaplegic homolog3	P84022	15
SMAD4	Mothers against decapentaplegic homolog 4	Q13485	18
MYC	Myc proto-oncogene protein	P01106	8
Rho	Rho-related GTP-binding protein Rho6	Q92730	3
CyclinD1	CyclinD1	Q9H014	11
P53	Cellular tumor antigen p53	P04637	17
Bcl2	Apoptosis regulator Bcl-2	P10415	18
Bax	Apoptosis regulator BAX	Q07812	19
KRAS	GTPase KRas	P01116	12
CASP3	Caspase-3	P42574	4
CASP9	Caspase-9	P55211	1
TCF	Transcription factor 7	P36402	20
LEF	Lymphoid enhancer-binding factor 1	Q9UJU2	4
JUN	Transcription factor AP-1	P05412	1
TGFR1	TGF-beta receptor type-1	P36897	9
TGFR2	TGF-beta receptor type-2	P37173	3
P13K	Phosphatidylinositol 3-kinase regulatory subunit alpha	P27986	17
Raf	RAF proto-oncogene serine/threonine-protein kinase	P04049	3

**Figure 1:**
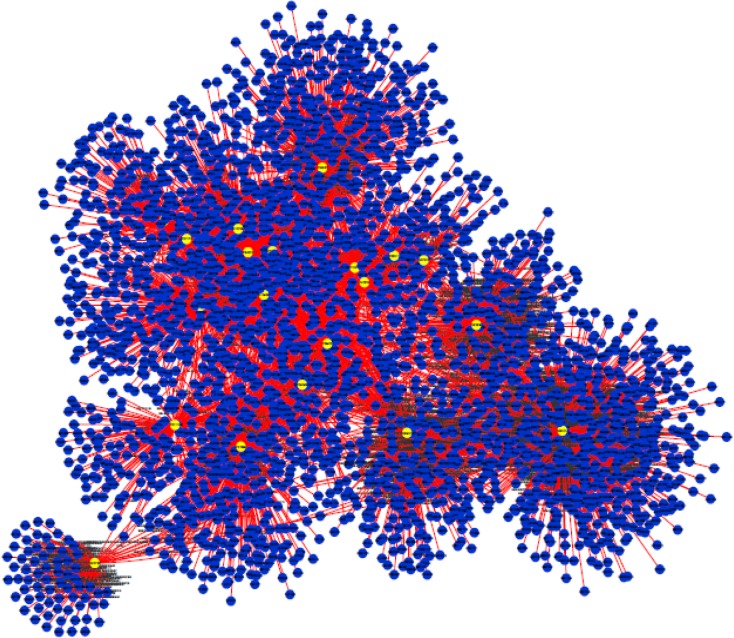
This PPI network consists of 2916 nodes and 5791edges. The highlighted nodes indicate the designated genes for the study. Based on degree and betweenness centrality, P53, CyclinD1, P13K, LEF1, BAD, KRAS, JUN, SMAD4, SMAD3, RAF1, MYC possesses significant centrality values.

**Figure 2:**
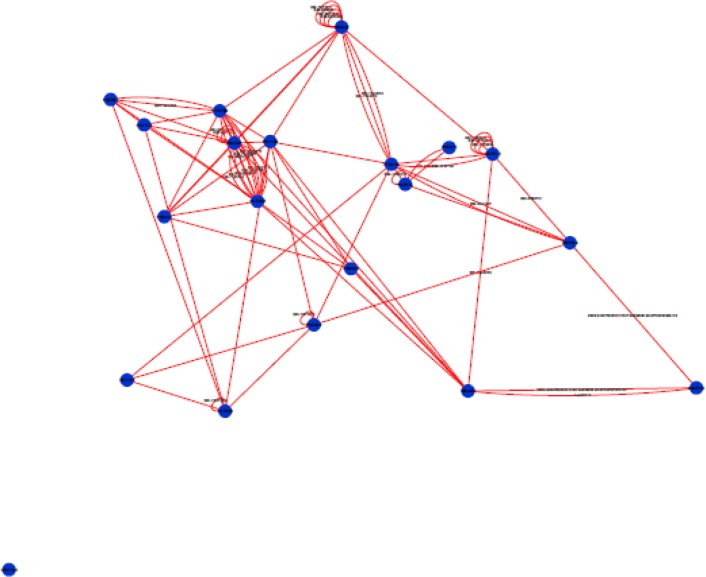
A number of nineteen Smad2 genes are connected directly except Rho gene.

There are many references that contain genes related to the breast and colon cancer; however, only common genes from pathway analysis were chosen. Twenty common genes were identified between breast and colon cancer through pathway comparison (table 1). In addition, for similarity confirmation between breast and colon cancer, pathways of basal skin cancer and thyroid cancer were also compared with the neoplasm of breast and colon. However, only eight common genes were identified between thyroid cancer and the designated cancers. In basal skin cancer pathway, only four genes were observed common with colon cancer and three with breast cancer pathways. 

Protein-protein interaction analysis was applied for network structure and function relationship study (27). The integrated network was obtained from MINT, Reactome-Fls, and STRING databases by the application of Proteomics Standard Initiative Common QUery InterfaCe (PSICQUIC) source (figure1). Figure 2 shows a topological view of direct interactions of the selected genes.

**Figure 3:**
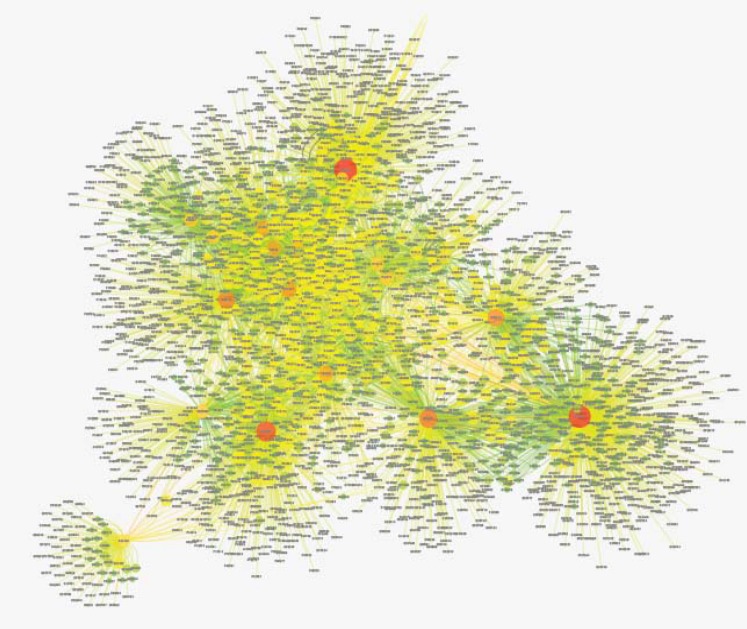
Mapping degree parameter of candidate proteins interaction network to node size and color. The visualization resulted using Network Analyzer plug-in. As the color of nodes change from green to red and the size of the nodes get bigger, the parameter value increases. The betweenness centrally data is not shown.

The topological analysis is important to characterize the biological value of genes (19). Different topological parameters can be evaluated through Network Analyzer. Here, analysis of betweenness and degree are handled by Network Analyzer (figure 3).

**Table 2:** A number of twelve genes with significant centrality value derived from figure 3, based on two fundamental centrality properties analysis (Degree and Betweeness centrality).

**Gene name**	**Degree**	**Betweenness centrally**
P53	813	0.23
Cyclin D1	761	0.31
P13K	591	0.19
LEF1	436	0.16
JUN	398	0.14
BAD	357	0.12
Smad4	330	0.09
Smad3	298	0.07
Smad2	264	0.1
RAF	258	0.06
MYC	215	0.07
KRAS	164	0.06

**Figure 4:**
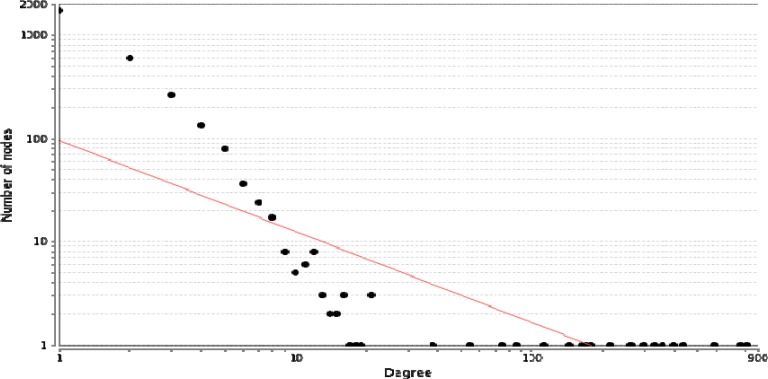
A scale-free network. The degree distribution is significantly inhomogeneous. Just a few nodes show well linked, whereas others possess a small numbers of connections. This distribution implies on the presents of proteins with high centrality values computed by Network Analyzer. The red line indicates the power law.  The R-squared value is computed on logarithmized values which is equal to 0.652 and the correlation= 0.9. Genes with high degree are in the right down region of the plot (their location is out of linear range).

Degree distribution of the complex PPI network can be obtained by Network Analyzer (24). Node distribution based on degree confirms the presence of genes with high centrality values (figure 4). 

**Table 3:** MCODE algorithm analysis demonstrates 5 clusters based on the number of interconnections in the large network of protein-protein interactions.

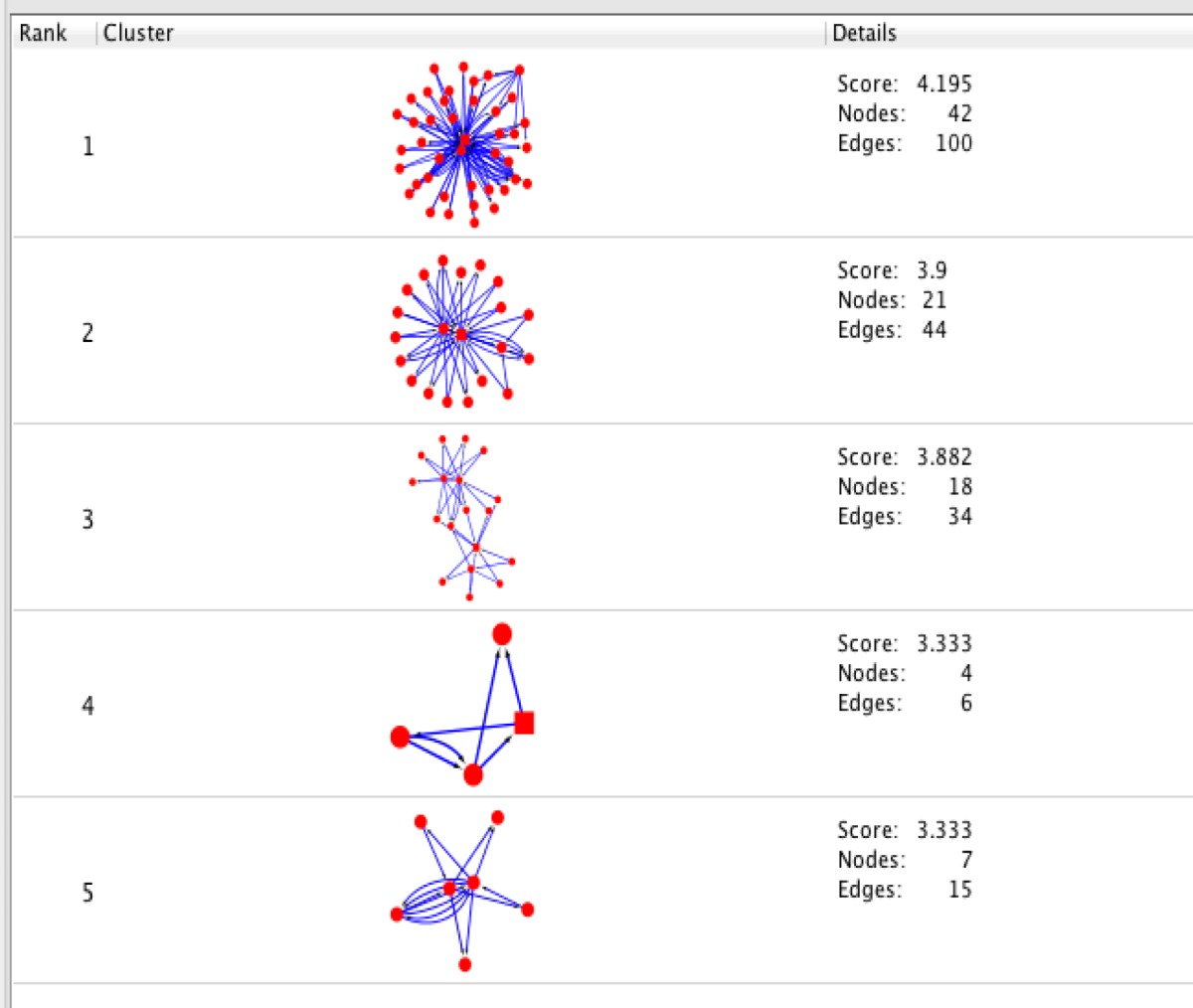

Among twenty genes, some belong to these clusters. Cluster1 is the highest ranked cluster, this protein complex assigned with 4.195 score. Candidate genes in the clusters are as follow: Cluster1: TCF, SMAD2, SMAD3. Cluster2: SMAD4, MYC, Bcl2 Cluster 3: KRAS, TGFR1, TGFR2, JUN Cluster4: BAD, Bax, Cluster 5: p53, P13K. Node Score Cutoff: 0.2, K-Core (a graph of minimal degree *k)*: 2, Threshold: 2 Cluster Score= Density*#Nodes.

As depicted in figures 3 and 4, based on node color and size as well as data computation, it can be inferred that there are twelve genes significant genes in the network (table 2).

**Table 4:** A list of seed nodes of clusters 1 to 5 (Cluster 5, does not contain any seed). As it is clear from table 3, no square is noticed for cluster 5. In addition, the highest score belongs to cluster 5 is two, that there are more than one node with this value. Seeds are SMAD2, SMAD4, JUN and Bax.

**Cluster No**	**Gene name**	**Score**
1	Smad2	4.46
2	Smad4	5.85
3	JUN	3.09
4	BAX	3.4

**Figure 5:**
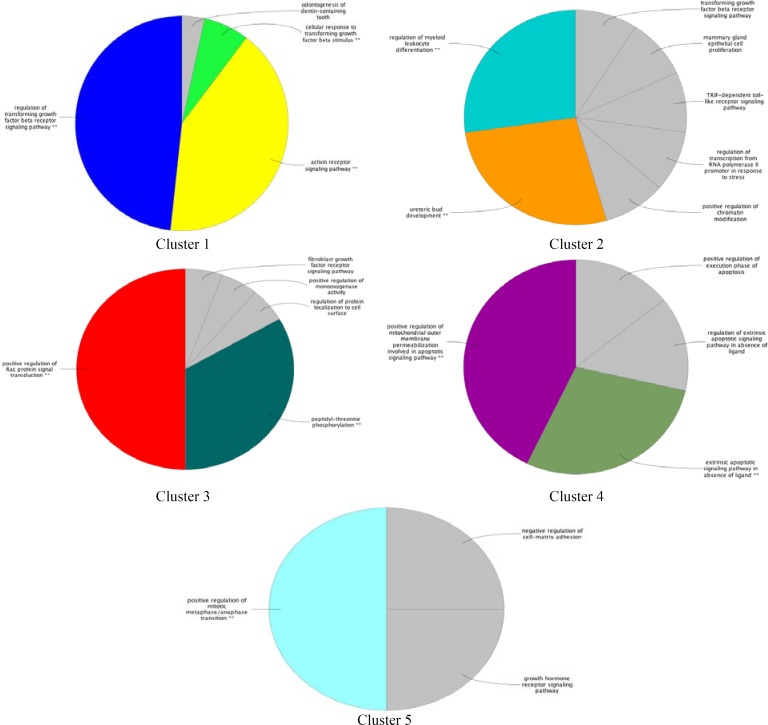
Functional distribution of biological process of modules of breast and colon neoplasm. (*P*<0.05). Clusters1,2,3,4,5 are significantly enriched in regulation of transforming growth factor beta receptor signaling pathway (10 terms), (regulation of myeloid leukocyte differentiation (3 terms), ureteric bud development (2 terms)), positive regulation of Rac protein signal transduction (7 terms), positive regulation of mitochondrial outer membrane permeabilization involved in apoptotic signaling pathway (3 terms), and positive regulation of mitotic metaphase/anaphase transition respectively (2 terms). In addition, some of the important proteins are involved in the mentioned notable terms. These include SMAD2, SMAD3 that are corresponded to regulation of transforming growth factor beta receptor signaling pathway, MYC belongs to regulation of myeloid leukocyte differentiation and ureteric bud development and Smad4 related to ureteric bud development. TGFR1 and TGFR2 are associated to positive regulation of Rac protein signal transduction, BAD, Bax proteins are relevant to positive regulation of mitochondrial outer membrane permeabilization involved in apoptotic signaling pathway. P53 is distributed to positive regulation of mitotic metaphase/anaphase transition.

MCODE is a clustering algorithm that determines modules (highly interconnected regions of proteins). The constructed modules are based on connectivity data derived from the PPI network in our study. Seed nodes are assigned with the highest score in a specific cluster. Scoring is computed by weighting each node and its neighbors (28). Nodes, whose scores are above the threshold, are selected for a specific cluster. Cluster members are colored red and square nodes represent seeds (table 3). A list of determined seed genes and their scores are tabulated in the table 4. ClueGo allows gene annotation based on three GO terms. Here, assessment of biological process for the five subnetworks with the cutoff of *P*<0.05 was performed. The lowest probability (*P*<0.001) showed with two stars. ClueGo integrates similar terms of a group with their associated genes for redundancy reduction. The largest area of the chart reflects the most significant group and the label reflects the group leading term. The terms with no significance are colored gray (figure 5). 

## Discussion

As many genes are common among breast and colon cancer, it is important to evaluate the possible relationship. There are many genes that are involved in the breast and colon cancer pathogenicity (2, 5), but only part of them are included in databases and databanks. In this study the proteins included in KEGG Pathway and WIKI Pathways are considered for analysis. According to literature survey, more than 40 highly reported proteins among breast cancer and colon cancer are common. More than 100 articles from Google Scholar were reviewed for biomarker comparison. However, considering reality and validity, there are only twenty of them (genes) are involved in the studied pathways. Whether this high rate of molecular distribution, can be interpreted as a close relationship between these two malignancies or not it requires more investigations. Comparison of other types of cancers (skin and thyroid cancer) with cancer of breast and colon was performed for this reason. However, only a limited number of genes were indicated frequent. The contribution of many common molecular agents in breast and colon cancer imply on performing in depth investigation of these common features. First of all, this analysis can lead to decipher a better understanding of the etiology of both cancers. Second, this finding can improve the sensitivity and specificity of diagnostic methods for breast and colon cancer. Third, it can provide more epidemiological evidences for incidence of these two cancers. In this paper, interference of breast and colon cancer, as well as a close incident of them is investigated. Through applied analysis, it is feasible to bring out a better resolution of molecular aspects of these diseases. In a way that, the evaluation of significant related genes can be useful for biological characteristics and molecular interactions. PPI network is the basic skeleton for proteins to handle their functions in the terms of the self-organization and homeostasis in biological system Aberrant function of each molecular agent in the network, can enhance the risk of many diseases such as cancer (30). In this study, protein-protein interaction analysis indicates the integrated network of designated genes. 

In fact, these genes are in close interactions. As it is depicted in figure 1, the twenty common genes (table 1) form a connected network; however, only one of them is not (Rho) connected directly to the others (figure 2). Scale free network of candidate proteins comprises of heterogeneous nodes based on different amounts of degrees (figures 3 and 4). Hub genes are important to study due to their centrality role in a PPI network. Deletion of hub genes can lead to vast fatal impact on the integrity of the biological network (20). A total of twelve genes (figure 4 and table 2) show high functional properties including: P53, CyclinD1, P13K, LEF1, BAD, KRAS, JUN, SMAD4, SMAD3, RAF1, and MYC. The role of each of these proteins in different types of cancers is reported in the numerous references (5, 10, 19, 31-34). However, these genes are presented as an identified panel in this study. Among them, P53, CyclinD1, and P13K are the most significant ones in centrality value. The P53 is an important protein in different types of cancers. In fact, changes in p53 gene can impose vast alteration in cell function (35). There is a great over-expression of this protein in human breast and colon cancer (36, 37). Cyclin D1 has a prominent role in cell cycle regulation. It is normally up-regulated protein in these cancers (38, 39).  The pathway of PI3K–Akt plays an important role in the cancer onset and development (40-42). Furthermore, these three proteins have a major role in many cancer types (40, 43, 44). The other hub proteins are also involved in different types of cancers. Since the investigated proteins are in close interactions, identifying protein complexes can provide another level of molecular insight. MOCDE clustering algorithm (center-based) demonstrates the presence of molecular complexes in this PPI network with possible similar functional properties (table 3). These complexes contain some of introduced valuable genes with considerable interactions. The first subgraph with 42 genes contains three of twenty common genes, including Smad2, Smad3 and TCF. Among them, Smad2 is the seed with the high centrality value. Moreover, the cluster1 is the most significant interacted complex of proteins in the PPI network and it can be suggested as hub module. The second cluster with 21 genes has the distribution of other three high centrality genes, including Smad4, MYC and LEF1. Smad4 is the seed and is characterized with high centrality association. The third cluster possesses eighteen genes, in which four of them belong to our studied genes. The four genes include: JUN, TGFR1, TGFR2 and KRAS. Among them, JUN is the seed and considered as a high centrality node. In cluster4, BAD and BAX are our studied genes. BAX is the seed, but with no centrality importance. For cluster 5 with seven genes, P53 and P13K are the detected ones, which are also showing high centrality scores. The seed nodes and their corresponding clusters, as well as their scores are tabulated in table 4. These informative findings can be considered as common aspects of both cancers and are valuable in determining drug targets. Furthermore, more information about roles of five introduced clusters in the biological processes is presented in figure 5. These findings improve details about the mechanism of the both cancers. ClueGo provided functional annotation (BP) of the studied modules. Indicating that SMAD2 and SMAD4 as a significant centrality and seed elements are also distributed in the highest significant terms. Moreover, other genes with high amount of the degree and betweenness centrality, including SMAD3, MYC, BAD, P53 are also enriched in the notable studied terms. In addition, JUN is another gene with considerable value of module interconnection and centrality; however, not detected in groups of cluster 3 terms. SMAD2, SMAD3, SMAD4, MYC, BAD and P53 show another level of significance and consequently interpreted as essential hubs. Expression alteration of each hub genes (especially those with higher scores), may conclude to malfunction of many other genes and influence the integrity of PPI network. Diagnosis and therapeutic monitoring can be accelerated by targeting the specific hub genes. Introducing a common panel including twenty biomarkers for breast and colon cancer indicates that these two cancers may be originated from same etiological origin. Although, these twenty genes are characterized as related cancer genes in different types of cancers, their association as a unique panel for breast and colon cancer is a valuable tool for therapeutic aspects of diseases. It is reported that the incidence of colorectal cancer in aged female is higher than men (35). In the other study, findings correspond to at least two times stronger role of screening intervention in men than women (36). Similarity of breast and colon cancer etiology implies on the equation of the risk of these two malignancies. Therefore, it can be concluded that breast cancer in young women effects on the rate of colon cancer incidence. Thus, colorectal cancer incidence in aged women is higher than men.

 This study suggests that there is a close molecular relationship between cancers of the breast and colon. A similar panel of biomarkers for these two malignancies and therefore equal probability for incidence of the two diseases can effect on their epidemiology. In conclusion, the incidence rate of colorectal cancer in women due to competition with breast cancer is an aged depended phenomenon.
